# Regulatory Frameworks for the Access and Use of Genetic Resources in Latin America

**DOI:** 10.1007/s13744-022-01017-x

**Published:** 2023-02-02

**Authors:** Yelitza Coromoto Colmenarez, David Smith, Guillermo Cabrera Walsh, Andrés France, Natalia Corniani, Carlos Vásquez

**Affiliations:** 1 CABI Latin America, Botucatu, São Paulo Brazil; 2 CABI UK, Egham, Surrey UK; 3 FuEDEI, Hurlingham, Buenos Aires Argentina; 4 INIA Quilamapu, Chillán, Región del Biobío Chile; 5 Facultad de Ciencias Agropecuarias, Universidad Técnica de Ambato, Cevallos, Provincia de Tungurahua Ecuador

**Keywords:** Biological control, access and benefit sharing, Nagoya Protocol, genetic resources, digital sequence information

## Abstract

The Nagoya Protocol is a legal framework focused on the Access and Benefit Sharing of genetic resources, including Biological Control Agents. In order to comply with the Nagoya Protocol, countries in Latin America are establishing legal frameworks for access to genetic resources. Scientists face the challenges of the bureaucratic and administrative burden to obtain the access permits to study the biodiversity present in Latin American countries, which include the evaluation of biological control agents that can be used in sustainable production programs. In order to avoid the demotivation of scientists and students to work on biological control by blocking the opportunities to get new bioproducts, it is important to increase the communication between the regulatory authorities and the scientific community, to ensure the establishment of an effective structure and mechanisms to facilitate the process and reduce the time needed to obtain the access permits. On the other hand, the establishment of regional platforms for the exchange of information and harmonization of procedures can contribute to reinforce the collaboration among Latin American countries and facilitate regional studies and biocontrol activities. In this article, the legal framework in place in different countries in Latin America will be discussed and some possible solutions and ways forward to the major challenges observed will be presented.

## Introduction

**Fig. 1:**
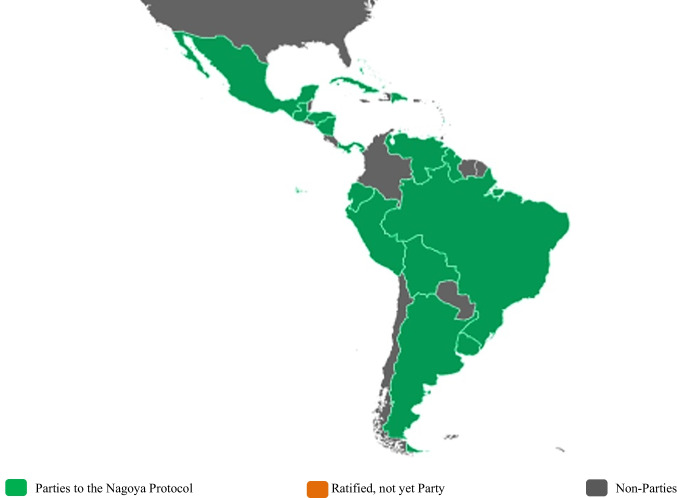
Parties (19) to the Nagoya Protocol from Latin America and The Caribbean from October 2014 to 30 March 2022. Access and Benefit-Sharing Clearing-House (2022)

The attack of pests and diseases represent one of the main limitations for agricultural production in the Neotropical region. It is estimated that at least 40% of all crops are lost in the pre- and post-harvest phases due to pests and diseases (Oerke and Dehne 2004). Within the sustainable control measures applied in Latin America, in the context of Integrated Pest Management (IPM), the use of biological control agents plays an important role (Van Lenteren et al. 2020, Santos et al. 2018; Colmenarez et al. 2016; Bergvinson 2004). Biological control in Latin America and the Caribbean has very favorable conditions due the rich biodiversity, resulting in significant numbers of species acting as natural enemies of pests, represented by parasitoids, predators, and entomopathogens (Alves et al. 2008). Research to evaluate biological control agents requires, in many cases, the access to resident natural enemies or importing-exporting species among different countries (classical biological control). In the past, biological control researchers and students used to access and exchange biological resources without facing too many complications or legal requirements. The implementation of biological control programs and the use of biological control agents are internationally regulated to ensure proper management of biodiversity (Aragón et al. 2018). In the last 20 years, Latin American countries have established legal frameworks to comply with the ISPM 3 of the International Plant Protection Convention (FAO 2005) and the Nagoya Protocol which is an agreement linked to the Convention on Biological Diversity and came into force in October 2014 (Fig. 1, Table 1). The Nagoya Protocol provides a legal framework for fair and equitable access to genetic resources, including biological control agents and potentially, the digital sequence information (DSI) generated from them, and sharing the benefits resulting from their use (Secretariat of the Convention on Biological Diversity 2011). DSI is a term used particularly in the Convention on Biological Diversity to refer to data derived from genetic resources (Cowell et al. 2022).

**Table 1:** List of parties (19) and non-parties (14) countries from Latin America and The Caribbean. Access and Benefit-Sharing Clearing-House (2022)

Country	Party from	National focal point (NFP)	Competent national authority (CAN)
Antigua and Barbuda	12 March, 2017	1	1
Argentina	09 March, 2017	1	1
Bahamas	30 March, 2022	1	0
Bolivia (Plurinational State of)	04 Jan, 2017	1	0
Brazil	02 June, 2021	1	1
Cuba	16 December, 2015	1	0
Dominican Republic	11 February, 2015	1	2
Ecuador	19 December, 2017	1	4
Guatemala	12 October, 2014	1	1
Guyana	12 October, 2014	1	1
Honduras	12 October, 2014	1	1
Mexico	12 October, 2014	1	6
Nicaragua	10 September, 2020	0	0
Panama	12 October, 2014	1	1
Peru	12 October, 2014	1	6
Saint Kitts and Nevis	04 December, 2018	1	1
Saint Lucia	12 June, 2022	1	0
Uruguay	12 October, 2014	1	1
Venezuela (Bolivarian Republic of)	08 January, 2019	1	1
Barbados	Non-party	1	0
Belize	Non-party	0	0
Chile	Non-party	1	0
Colombia	Non-party	1	1
Costa Rica	Non-party	1	1
Dominica	Non-party	1	0
El Salvador	Non-party	0	0
Grenada	Non-party	1	1
Haiti	Non-party	1	0
Jamaica	Non-party	1	0
Paraguay	Non-party	0	0
Saint Vincent and the Grenadines	Non-party	1	0
Suriname	Non-party	0	0
Trinidad and Tobago	Non-party	0	0

The Convention on Biological Diversity (CBD) is the international legal instrument for “the conservation of biological diversity, the sustainable use of its components and the fair and equitable sharing of the benefits arising out of the utilization of genetic resources” that has been ratified by 196 nations (United Nations 1992). The CBD agreement establishes the sovereign rights of each country on the genetic resources within its territorial borders. Likewise, it establishes that agreements that govern access to genetic resources and the sharing of benefits resulting from the use of the genetic resources must be established among interested countries (Cock et al. 2010).

It is well known by research institutions and private enterprises that research activities, use, production, importation, and export of biological products are governed by the legal framework applicable in each country. However, the applicability of the legal framework on access to genetic resources for research and development of biological products implies in many cases complicated-bureaucratic processes, which may limit scientists in the exchange of natural enemies for taxonomic and study purposes. In this article, the legal framework in place in different countries in Latin America will be discussed and some possible solutions and ways forward to the major challenges observed will be presented.

## The Nagoya Protocol and Related Regulations Concerning the Use of Biological Control Agents: Cases Studies—Countries Review

The implementation of legal frameworks for the management and research on biological control agents, which in some countries are rather complex for scientists to comply with, is considered to be a difficult task for many scientists. The major concern is the fact that the new biodiversity laws in Latin America can negatively affect the research and use of biological control agents, limiting the countries in the region to develop and improve sustainable pest management through the use of natural enemies (Coutinot et al. 2013; Silvestri 2017). In this regard, one of the main problems in Latin America and the Caribbean is that legal requirements change depending on the country and in some cases discourages the researchers from exchanging biological material, for taxonomic purposes, or studies on biological control agents, due to the time and bureaucracy involved.

In addition to controls on the genetic material itself, the biological control agent, the CBD Conference of the Parties, are negotiating on how DSI is treated. Already some countries include access and use in their regulatory controls; others have decided it is outside the scope of their ABS measures but despite this, options are on the table for negotiations and already the Open-Ended Working Group on the Post-2020 Global Biodiversity Framework are discussing it with a view to make recommendations to the 27th Climate Change Conference of the Parties–COP 27, implemented in Egypt in 2022 (OEWG 2022). A fact-finding study reports on several countries that have taken measures including in Latin America (AHTEG 2020). These countries include Brazil, Colombia, Costa Rica, Panama, and Peru. This adds additional complication and potential restriction on the use and characterization of biocontrol agents.

As suggested by Bergvinson (2004) and Colmenarez et al. (2016), some of the legal requirements for the exchange and use of biocontrol agents should be coordinated in Latin America and in the Caribbean through key organizations (e.g., COSAVE, CARICOM, OIRSA, FAO, among others), encouraging the exchange of materials and information between countries and finding common points in the legislation of each country to facilitate the process. Some examples of the legal frameworks and procedures in different countries in Latin America are discussed below.

### Argentina

In Argentina, the Nagoya Protocol was approved by Law 27.246 on December 9, 2016, and ratified on March 9, 2017 (Devia 2020; Access and Benefit-Sharing Clearing House 2022). Argentina issued Resolution 410/2019, which establishes the legal requirements to obtain authorization on access to genetic resources. The Ministry of Environment and Sustainable Development was designated as the ABS National Authority responsible for the supervision and implementation of the Nagoya Protocol and to provide the authorization to access the genetic resources at national level (Devia 2020). The issue of collecting, transporting, and exporting scientific samples and specimens in and out of Argentina has had many developments in the last 10 or 15 years that have resulted in a very complicated and restrictive system. Even though this tendency can be observed throughout the world, legislation in Argentina gives it particularly convoluted facets that often lead to jurisdictional deadlocks, making it extremely difficult and slow for scientists, local and foreign alike, to access and share scientific material (Silvestri et al. 2020). Silvestri (2017) highlighted some of the major challenges that the scientific community in Argentina was faced with the implementation of the Nagoya Protocol, due the complexity of the content, and the need to establish a multidisciplinary team to oversee the implementation of the legal framework. In Argentina, each province can adapt the legal framework locally, increasing the complexity. Silvestri et al. (2020) and Barratt et al. (2021) illustrate the complexity of the Argentinean framework by describing the steps involved in obtaining collection and export permits. The National Law No. 24375 and 13 other laws and decrees regulate the use of biological control agents (e.g., Resolution No. 410/19 that regulates access to genetic resources). The various provincial and national legislations essentially reflect the international agreements derived from the Convention on Biological Diversity signed at the 1992 Rio Earth Summit, including the Nagoya Protocol on Access to Genetic Resources and the Fair and Equitable Sharing of Benefits (ABS). However, complications arise from the very slow evaluation periods, incompatible with the timeframes of most scientific endeavors. When the Argentine Constitution was reformed in 1994, natural and cultural resources were placed under the jurisdiction of provincial authorities. The only exceptions are those located in the areas under jurisdiction of the National Parks Administration (APN), namely National Parks, Reserves, and Monuments. This means that only the provincial environment authorities, or national parks when applicable, can authorize the collection, shipping, and exchange of genetic material.

As a first step for international collaboration and exchange of scientific material, the foreign scientist must have a written agreement (a Material Transfer Agreement, or MTA) with a local scientist associated to a recognized institution (donor institution). Argentine law states that foreigners cannot access biological or historical/cultural samples (indigenous artifacts, fossils, etc.) on their own, as they must always have agreements with a local institution (donor institution/scientist). The foreign scientist must also be associated to a recognized institution (receptor institution). Similar conditions apply for exchanges between institutions of different provinces or districts (Plan de Acción Ministerio de Ambiente y Desarrollo Sostenible 2016–2020).

Requests for collection of scientific material must then be presented to the provincial or APN authorities by the donor institution. These authorities must provide the donor institution/scientist with (1) a collection permit, and (2) a transit permit should this material leave the province—or National Park. In order to export any part of the material, scientists must also obtain a (3) letter of consent from these authorities. This letter of consent certifies that the owner of the material (the province or the APN) is confident that the ABS terms of the MTA are acceptable and adjust to their regulations. Rules to obtain these documents vary from province to province, but they for the most part require the local and foreign institution to formulate (4) an MTA, and a (5) research project (Silvestri et al. 2020).

The format and terms of the MTA are usually not specified, and examples or templates are rarely provided, which further complicates and delays the process, since the scientists involved can never be sure if the provincial authorities will find the signed MTA, or the project, acceptable. Normally, these documents are expected to be similar to those used in most countries, and state the objectives and reach of the agreement, scientists and authorities involved, identity and number of specimens to be collected, explicit use of the material, validity term, statement that the species are not on a protected list, and a commitment to provide voucher specimens and periodical advance reports (Silvestri et al. 2020). Once the provincial or APN authorities approve this MTA, another (6) MTA is frequently requested to be signed between the owner of the material and the requesting party (donor institution).

Once the donor institution has the ABS consent, and the collection and transport permits, the scientists involved are in a position to proceed with their collections and research. However, if part of the material is meant to be exported out of the country, the scientist or institutional representative must request two permits from the National Ministry of Environment and Sustainable Development (MAyDS). The permits to export the material are issued by the (7) department of Flora and Fauna of the MAyDS. For this, the donor must present the ABS consent, and the collection and transport permits, copies of the signed MTAs, and project proposal. This permit assumes that biocontrol agents are a material that will be used as is, not manipulated genetically, and as such that the material does not fall under the provisions of the Nagoya Protocol. Under some circumstances, however, a second permit (8) is issued by the Biodiversity Conservation Group of the MAyDS. This is for the cases that are considered to be covered by the Nagoya Protocol and involves the material that will undergo molecular and/or genetic studies or use. In other words, when genetic units, or other molecules that can be considered units of inheritance, are to be studied or used (Resolution 610/2019). Note must be taken that not all provinces agree that organisms that will not be subject to genetic studies or manipulation should be exempt from Nagoya Protocol considerations. Some of the provinces consider that any organism, or any part of it, falls under ABS guidelines, and they make it explicit in the collection permits and/or ABS consent (Mc Kay, F., pers. comm.).

Once permit (7)—and (8), if required—are obtained, the (9) final export permit is provided by The National Food Safety and Quality Service (SENASA). This permit is subject to the presentation of permits (7) and (8), and (10) the importation permits from the relevant Inspection Service of the receiving country. Shipping of these samples can be done personally—hand-carried—by a scientist—or any other authorized carrier—subject to an express authorization by SENASA, although shipping brokers are preferred by local authorities. Notwithstanding this preference, experience demonstrates that hand-carrying is by far the best system to take living material, because brokers usually take up to a week to deliver material that can take less than 12 h if hand-carried. Furthermore, costs are not significantly greater, even counting all the carrier’s expenses and tickets. Also, international couriers, such as FedEx, UPS, and DHL, as well as many airlines, usually refuse to ship any type of biologicals from Argentina, and even when they accept the shipment, they cannot be trusted to deliver within reasonable timeframes. Preserved samples do not normally have these problems.

In all this 10-step process, the intervention of the MAyDS and SENASA is not usually problematic and is normally resolved within 3 to 6 weeks. By far the slowest and most problematic steps are the provincial permits and agreements, which have been known to take months or years to complete (Silvestri et al. 2020) or are refused flatly on arbitrary or misinformed grounds.

On an up note, MAyDS is working as from 2017, on a Federal Congress law for the preservation and conservation of biodiversity that should force provincial and national departments to unify procedures and criteria. There is currently no deadline for the establishment and enactment of said law.

To date, Argentina does not control DSI in their legislative or policy measures (https://absch.cbd.int/en/) but they are considering including it and it is possible that authorities will consider it equal to actual genetic material.

### Brazil

Brazil has been a signatory country of the United Nations Convention on Biodiversity since 1992 (CBD 1992) and it has invested substantial resources to study and conserve biodiversity even before ratifying the Nagoya Protocol (Alves et al. 2018; Peixoto et al. 2006). Brazil ratified the Nagoya Protocol on March 4, 2021. The Genetic Heritage Management Council (CGen) has a central role on managing the information that is the core of the ABS compliance (Davis et al 2016). In order to enable compliance with the legislation, the National System of Genetic Resource Management and Associated Traditional Knowledge (SisGen) was developed by the Ministry of Environment (da Silva 2019). In Brazil, scientists need to follow the legal requirement when working on Biological Control which implies the use and access to biological resources or the devolvement of biological products; in all cases, they should comply with the procedures established by the government in the Law 13–123, of May 20, 2015 (Brasil 2015). It is important to note that the requirements contained in Law 13,123 should be observed regardless of the date of collection of the samples or the form of their acquisition. Brazil has advanced in refining the legislation in place and is one of a few countries in Latin America that includes genetic information in the scope of its ABS legislation (da Silva 2019). Despite the issue of DSI has been discussed in the last two meetings of the Conference of the Parties to the CBD (COP 13 and COP 14) and of the Parties to the NP (COP-MOP 2 and COP-MOP 3), not many countries in Latin America and the Caribbean have included DSI as part of the ABS requirements as yet.

The registration of the research activity or technological development shall precede any of the following actions:

I Submission of samples abroad
II Request for an intellectual property right over the product of access
III Notification to the Genetic Heritage Management Council “*Conselho de Gestão do Patrimônio Genético*” (CGEN) of the finished product or the reproductive material developed
IV Marketing of intermediate product
V Dissemination of results, final or partial

The Law No. 13–123, of 2015, establishes special rules for the regularization of activities, and for the regularization of access to genetic heritage for scientific research purposes, the user registers or obtains authorization from the CGEN, following the rules established by the law itself (Brasil 2015). It is important to highlight that regularization is, for a very specific situation, only related to the non-compliance with previous legislation, as the Law provides that regularization will be required for any activity that was performed contrary to the Provisional Act 2,186–16, of August 23, 2001, and within the scope of this legislation (da Silva and Oliveira 2018).

In accordance with the definitions of the Provisional Measure No. 2,186–16 (Brazil 2001), for the regularization of bioprospection and development activities and economic exploitation of product or process, the interested party must sign the Commitment Term with the Union, which shall provide the following (Vasconcelos 2016):

I The request for the access authorization or consignment, registering the activity, following the rules established by Law No. 13–123 (Brasil 2015)
II Notify the product or process coming from the access
III Distribute, in accordance with the new rules established by Law no. 13–123 (Brasil 2015), the benefits arising from the economic process or product from the access within maximum of 5 years after signing the Commitment Term

In terms of accessing microorganisms, in Brazil, an isolate from an ex situ collection that was obtained pre-CBD would be in scope of their national legislation (article 2 from the Law 13.123/2015) and the Nagoya Protocol (Smith et al. 2017).

Coutinot et al. (2013) highlighted how complicated and diverse the steps are to be followed to obtain the required permits for access and exportation/importation of natural enemies in Brazil. The authors highlighted the fact that biological control agents are living organisms and unmodified genetic resources that cannot be patented and should not follow the same treatment given to commercial products (e.g., drugs, seeds). According to the authors, during the last years, the biological control specialists have faced complicated legislation established by a high and diverse number of governmental agencies, making the access to natural resources for biocontrol purposes a rocky road. However, in the last couple of years, communication between the Brazilian Government agencies has tended to be more efficient, establishing a more harmonized and less complicated way of working with the scientific community making sure all scientists know the procedures for the application processes well (Vasconcelos 2016).

Part of the Brazilian Agricultural Research Corporation (EMBRAPA), in the municipality of Jaguariúna, state of São Paulo, is located the “Costa Lima” Quarantine Laboratory (LQCL), for Monitoring and Environmental Impact Assessment (CNPMA). This is the only Brazilian quarantine facility authorized by the Ministry of Agriculture, Livestock and Food Supply (MAPA) to introduce natural enemies for pest control, as well as other beneficial organisms for scientific research, as established by Decree 106 (dated of June14, 1991). Therefore, in order to provide the necessary requirements for the movement of natural resources, the LQCL provides the following services: (1) elaboration of documents required for the importation petition form requested by the Ministry of Agriculture (http://sistemasweb.agricultura.gov.br/sislegis/action/detalhaAto.do?method=visualizarAtoPortalMapa&chave=1136368974); (2) supply of the information (collected locally and evaluation of available organisms) concerning importation of natural enemies; (3) provision of quarantine services focused on the security for the introduction of exotic organisms; (4) maintaining of documents on the imported organisms, as well as the voucher specimens at the LQCL Entomological Collection (Sá et al. 2002; Sá and Oliveira 2006).

Among its scope, LQCL must subsidize the Plant Sanitary Defence Coordination Office by providing technical advice and feedback about the requests for importation of exotic natural enemies for pest control in Brazil.

The procedures for exportation of natural enemies through the Brazilian Institute of Environment and Renewable Natural Resources of the Ministry of the Environment require the Convention on International Trade in Endangered Species of Wild Fauna and Flora-CITES form that can be accessed at http://www.ibama.gov.br/cites-e-comercio-exterior/licenca-cites/legislacao-licenca-cites#content.

Brazil’s Biodiversity Law (Law No. 13,123) requires registration of users and Mutually Agreed Terms (MAT). The law defines “genetic information from plants, animals, and microbial species, or any other species, including substances originating from the metabolism of these living organisms.” Thus, the inference that the scope of the law includes DSI is because of the “genetic information” present in the definition. Therefore, DSI must be negotiated within MAT when accessing and using Brazil’s genetic resources (AHTEG 2020). This is reinforced by the fact that the Decree 8772/2016, which regulates the law, and requires that the origin of the genetic heritage be informed, including when the source is in silico. Additionally, it is important to note that the MAT is only required when there is a product not when the genetic heritage, including DSI, is accessed. Besides that, the requirement of registering the use of genetic heritage is only before (a) request for an intellectual property right over the product of access; (b) notification to the Genetic Heritage Management Council “Conselho de Gestão do Patrimônio Genético” (CGEN) of the finished product or the reproductive material developed; (c) marketing of intermediate product; or (d) dissemination of results, final, or partial. Effectively, notification and MAT are required when there is a product placed on the market.

### Chile

Regarding the Nagoya Protocol, Chile has not ratified this agreement. There is also no legislation of its own that regulates access to local genetic resources, or a competent authority in the microbial domain, to which the regulatory framework on petitions can be requested from abroad or consultations on endemic or native microorganisms. However, some government agencies have established procedures and guidelines for access to genetic resources, focusing on germplasm banks and protected areas (UEBT 2020). In the absence of these protocols, the Chilean Microbial Genetic Resources Collection (CChRGM) (https://www.cchrgm.cl) has tried to contribute in this sense establishing a national record, as a public bank and accessible to all people inside and outside the country. Eventually, the CChRGM could act as a reference to public and private consultations related to the exchange and conservation of microorganisms, while regularizing a procedure at the national level. The Agricultural and Livestock Service (SAG) is the state agency responsible for protecting animal and plant health from the entry of pests and diseases into the national territory. Based on internationally recognized norms and procedures, the SAG regulates the exchange of biological material in Chile, especially those entering the country, to avoid any possible health problems, as well as controlling the institutions that receive this material (https://www.sag.gob.cl) (Barratt et al. 2021). The ABS National Focal Point is the Ministry of Environment (Traditional Knowledge National focal point/Global Strategy for Pant Conservation National Focal Point) (Access and Benefit-Sharing Clearing-House 2022).

Regarding the importation of biological products, the process is divided into those materials with no commercial value, for which the main purpose is only research, and those materials that are used for commercial purposes, e.g., industrial consumption or use, which require a more complex process to enter into the country. In the first case, resources are considered samples and require an import permit, whose form is evaluated by the SAG through a technical and risk analysis, prior to its approval. This form requires information about the following:

- Origin of the sample
- Purpose of the analysis
- Methods of destruction of sample remains
- Applicant’s background
- Place of entry into the country
- Description of the sample to be entered

The above form is designed for the entry of biological materials for research purposes that can self-reproduce, such as fungi, bacteria, nematodes, plants, and animals, or be reproduced, such as phytoplasmas, viruses, virions, and prions. Materials such as nucleic acids, antibodies, enzymes, milk cultures, cell lines, and histological sections are excepted. These permits are unique authorizations, which require a new form each time you want to enter a new material.

For other types of authorization of entry of biological materials and those that are required repetitively or as standards of comparison, there is another form called “Request for Import Permit of Samples of Drugs, Raw Materials, Strains and Analytical Standards,” which is aimed to be used by companies previously registered in the SAG as authorized manufacturers or importers.

The SAG is also responsible to establish the conditions and requirements to authorize microbial pesticides for commercialization within the country, having a new Law in force since December 2018. It determines the conditions for the importation of products of microbial origin and the validation protocols, prior to their approval as biopesticides (Barratt et al. 2021). Even though these regulations do not consider the exchange of material for scientific purposes, they facilitate the entry of biopesticides for efficacy trials as well as national microorganisms and bioproducts that may be exported.

In other aspects related to the exchange of biological material, Chile is a signatory to several international agreements that are related in some way to genetic resources and their commercial uses or to research, such as the following:

- International Treaty on Plant Genetic Resources for Food and Agriculture (FAO 2009)
- Convention on Biological Diversity (United Nations 1992)
- Organization for Economic Cooperation and Development (OECD 2009)
- International Treaty on Plant Genetic Resources for Agriculture and Food (FAO 2016)
- Budapest Treaty on the international recognition of the deposit of microorganisms for the purposes of the patent procedure (WIPO 2011)

Nowadays, the biggest challenge for the exchange of biological material is to have a regulation that facilitates the entry and exit of these materials, for research or commercial purposes. The internal pressures in which it is claimed that genetic resources and traditional knowledge of indigenous peoples could be privatized, if treaties like Nagoya are signed, are the biggest restriction for Chile to adopt international legislations and give legal clarity about the good use of these biological resources.

### Colombia

Colombia has not ratified the Nagoya Protocol yet but is a signatory of the protocol since Feb. 2, 2011 (Access and Benefit-Sharing Clearing-House 2022). Access to genetic resources worldwide is an important issue, not only because it ensures the safety of biodiversity but also because it recognizes the wealth of each nation. For Colombia as a Megadiverse country, genetic resources and their derived products are by law owned by the State and are therefore inalienable, imprescriptible, and non-releasable. The Resolution 1348 of 2014 modified by Resolution 1352 of 2017, issued by the Ministry of Environment and Sustainable Development for the proper application of Andean Decision 391 of 1996, establishes that natural or legal persons wishing to develop projects or research activities that involve access activities to genetic resources and/or their derived products must sign an “Access to Genetic Resources and Derived Products” (Rojas et al. 2021).

Although the country is not a Party to the Nagoya Protocol, the Office of Green and Sustainable Business has developed an internal procedure for the subscription of contracts on access to genetic resources and derived products (Rojas et al. 2021).

In order to advance the process of accessing the genetic resources and their derived products in the Ministry of Environment and Sustainable Development, it will have to identify if some of the activities indicated in Article 2 of Resolution 1348 of 2014 will be carried out or if it is applied in paragraph 3 of Resolution 1352 of 2017 issued by the Ministry of Environment and Sustainable Development, which establishes the following:

- Resolution 1348 of 2014, Article 2 “Activities that configure access to genetic resources and their derived products.” The following activities are considered to access genetic resources when are carried out with native species, whether wild, domesticated, cultivated, or domesticated, including viruses, viroids, and the like, that are located in the national territory or out of this:
  1. Those that claim the separation of the functional and non-functional units of the DNA and/or RNA, in all the forms that are in the nature.
  2. Those that claim the isolation of one or more molecules, understood as micro and macromolecules, produced by the metabolism of an organism.

Paragraph 2 establishes that it does not establish access to genetic resources and their derived products in addition to the provisions of paragraph 1 of article 4 of Decree 1375 and paragraph 5 of article 2 of Decree 1376 of 2013 (molecular systematics, molecular ecology, evolution, and biogeography), the activities indicated in this article that are carried out on genetic resources and products derived from species introduced into their wild, domesticated, cultivated, or domesticated escape and those of human origin.

In the Resolution 1352 of 2017, the Article 3 establishes that when a patent application is sought for products or processes obtained or developed from genetic resources or derived products, the applicant must submit to the competent national office a copy of the contract for access to genetic resources and their products derived in accordance with the provisions contemplated in Andean Decision 486 of 2000 (Rojas et al. 2021).

Once identified if the activities it intends to carry out, or not, access to genetic resources and their derived products, the application for a contract of access to genetic resources and their derived products may be initiated with the Ministry of Environment and Sustainable Development. This contract may be established for industrial, commercial, or biological prospecting purposes.

The following presents the step by step process for the preparation of contracts for access to genetic resources and their derived products in Colombia.

The contract can be requested by a natural or legal person for the development of a specific project; in this case, the applicant must complete all the information contained in the request format to access genetic resources and additionally must provide the following:

- The documents of existence and representation in case of being a legal person or the citizenship card in the case of being a natural person.
- Form of request for access to genetic resources due diligence.
- The letter where the National Support Institution (INA) commits itself as such under the terms set forth in Andean Decision 391 of 1996. This letter must be signed by the legal representative or by his designate or proxy; in the letter, the document that empowers the person to sign the communication must be attached.

The INA is a national legal entity different from the applicant; it may be public or private that is dedicated to the research and is willing to accompany the applicant in the investigation and support the Ministry of Environment and Sustainable Development in the follow-up activities of the obligations of the applicant, provided for in the contract the INA must be from Colombia. It is important to note that INA does not form part of the contract, nor is it obliged to respond to the applicant’s commitments:

- The resume of the responsible technician and the work team.
- Certification on the presence of ethnic communities in areas where species collection is planned, when this activity is contemplated within the research (it must be brought before the Ministry of the Interior).
  - If there are ethnic communities in the areas where the collection is to be carried out, the applicant must exhaust the process of prior consultation with the Ministry of the Interior and submit to the request for contract the record signed by the Ministry of the Interior once it reaches an agreement with the community.
  - If there are communities present in the collection areas, it will suffice to be certified by the Ministry of the Interior.
- If the samples are to be taken from a collection the applicant must provide a document certifying that the collection is registered with the Humboldt Institute and a statement by the curator of the collection indicating the donation of the samples for the development of the project.
- Authorization to work with LMOs or GMOs if applicable.
- Accessory contracts if any, as established in Article 41 Andean Decision 391 of 1996.
- If the project is for commercial purposes, a business plan, a market study, or a similar report should be presented to identify sales projections and production costs, a proposal for the distribution of monetary benefits by the applicant, and the information that it considers pertinent to be presented in the negotiation.
- In case of requesting confidentiality regarding some information of the project, it should be indicated in a communication, presenting the justification, and filling out the form of application for access to genetic resources with the respective non-confidential information (a summary that is of a public nature).

In case the researchers are not sure whether or not the activities they intend to conduct need the contract to access the genetic resource, detailed information about the research project, such as project title, objectives, methodology, and possible expected results need to be sent to the Ministry of Environment and sustainable development, which will determine if the research project requires the contract for access to genetic resources (Rojas et al. 2021).

For this process, the user should contact the Ministry of Environment and Sustainable Development, through the Genetic Resources Group of the Directorate of Forests, Biodiversity, and Ecosystem Services, who are in charge of managing in a comprehensive way the issue of access to genetic resources and their derivatives in Colombia and the fair and equitable distribution of benefits derived therefrom (Rojas et al. 2021).

Rojas (2013) discussed the challenges that researchers and students face to access biological resources to carry out a scientific investigation of biodiversity which can include the evaluation of the resident biological control agents. There have been various criticisms regarding the procedures and administrative processes presented. To conduct the studies, permits need to be obtained and must be requested to the environmental authorities according to the jurisdiction of the resource to be investigated; as a further step, a contract is signed to access natural resources and, in the case that the studied area is in the jurisdiction of indigenous peoples, prior informed consent must be obtained through consultation, which can delay the processes. Rojas (2013) highlights that in order to comply with the Nagoya Protocol, countries need to establish an administrative institutional mechanism and ensure it operates in an efficient way to minimize the bureaucracy and the administrative burden.

The development of the “Handbook for Access to Genetic Resources and their by-Products in Colombia” facilitates the familiarization with the legal framework in place to access the genetic resources and contributes to a better understanding of the procedures to follow as part of the requirements (Rojas et al. 2021).

Decision 391, Resolution 1348 of 2014 on access to genetic resources and its derivatives establishes that “functional units of heredity must be understood as those that contain the code for a gene.” As a result, it is possible to grant contracts for access to and the use of DSI and its corresponding benefit-sharing. Colombia considers DSI as an integral part of its genetic resources. If such use is intended for a Colombian native species, which could be found in public or private databases, a contract for access to genetic resources and its derivatives should be signed with the Ministerio de Ambiente y Desarrollo Sostenible and respective benefit-sharing with Colombia is expected.

### Costa Rica

Since 6 July 2011, Costa Rica is a signatory of the Nagoya Protocol but has not ratified it yet (https://www.chmcostarica.go.cr/sobre-la-cdb/protocolos). The National Biodiversity Law (No. 7788), formalized in 2015, includes among its objectives the reduction of threats that affect sustainable use and conservation of biodiversity, as well as establishing guidelines to help recognize its value. The National Commission for Biodiversity Management (CONAGEBIO) is the institution in charge of granting access permits for research projects, bioprospecting, and economic use. A virtual platform has been developed by CONAGEBIO, to request the access permits (http://www.conagebio.go.cr/Conagebio).

The country makes available the National Biodiversity Policy and Strategy (https://cidicer.so.ucr.ac.cr/sites/default/files/content/Ley%20de%20Biodiversidad.pdf) and a more accessible explanation of the Law in a virtual platform for its follow-up. (https://portals.iucn.org/library/sites/library/files/documents/2000-098.pdf).

The National Biodiversity Law requires the user to acquire a permit to access GR and DSI and establishes subsequent benefit sharing. Costa Rica considers that the analysis and use of DSI is a type of subsequent utilization of genetic or biochemical resources; therefore, it must be regulated. Furthermore, DSI is covered under the definition of “access to genetic resources” of the Biodiversity Law (the definition of access includes to obtain associated knowledge of the samples of biodiversity) (AHTEG 2020).

## Final Considerations

As discussed in Colmenarez et al. (2016) and Bergvinson (2004), it is important to develop a Regional Platform that allows the harmonization of the procedures, and facilitate the exchange of information among the countries. This will allow the development of new collaborative platforms on Biological Control. The governments have a fundamental role in the adoption of biological control, by promoting the use of biocontrol agents as an alternative to chemicals in Integrated Pest Management systems; in this way, it is important to establish legal frameworks and procedures that are well-known among scientists and feasible to be implemented in a reasonable time. In order to facilitates the harmonization of the procedures and the exchange of information and experiences when applying those in the different countries, in South America, the Plant Protection Committee of the Southern Cone (Comité de Sanidad Vegetal del Cono Sur–COSAVE), a Regional Plant Protection Organization, is considered to be a key player to develop a South American platform possible for harmonization of the biodiversity legal framework in the region. It is suggested that COSAVE creates the opportunity to have a multidisciplinary group from the different countries to develop a South American Platform for Legal issues associated to research and implementation of biocontrol programs in the region, as part of the activities carried out by the established Biological Control Working Group. Ideally, the establishment of multidisciplinary subcommittees could help organizing standards and protocols for Latin America and the Caribbean regulating the movement of beneficial organisms between countries. With joint efforts at the national and regional level, key institutions can make possible the use of Biological Control Agents, to a large extent, be strengthening sustainable production and food security in the region.

## Data Availability

Data sharing is not applicable to this article as no new data were created or analyzed in this study.
