# Correction: Regulatory Frameworks for the Access and Use of Genetic Resources in Latin America

**DOI:** 10.1007/s13744-024-01161-6

**Published:** 2024-04-29

**Authors:** Yelitza Coromoto Colmenarez, David Smith, Guillermo Cabrera Walsh, Andrés France, Natalia Corniani, Carlos Vásquez

**Affiliations:** 1 CABI Latin America, Botucatu, São Paulo Brazil; 2 CABI UK, Egham, Surrey UK; 3 FuEDEI, Hurlingham, Buenos Aires Argentina; 4 INIA Quilamapu, Chillán, Región del Biobío Chile; 5 Facultad de Ciencias Agropecuarias, Universidad Técnica de Ambato, Cevallos, Provincia de Tungurahua Ecuador

**Correction: Neotropical Entomology (2023) 52:333–344**

10.1007/s13744-022-01017-x

The article “Regulatory Frameworks for the Access and Use of Genetic Resources in Latin America”, written by Yelitza Coromoto Colmenarez, David Smith, Guillermo Cabrera Walsh, Andrés France, Natalia Corniani & Carlos Vásquez, was originally published Online First without Open Access. After publication in volume 52, issue 2, page 333–344, the author decided to opt for Open Choice and to make the article an Open Access publication. Therefore, the copyright of the aticle has been changed to © The Author(s) 2023 and the article is forthwith distributed under the terms of the Creative Commons Attribution 4.0 International License, which permits use, sharing, adaptation, distribution and reproduction in any medium or format, as long as you give appropriate credit to the original author(s) and the source, provide a link to the Creative Commons licence, and indicate if changes were made.

The images or other third party material in this article are included in the article’s Creative Commons licence, unless indicated otherwise in a credit line to the material. If material is not included in the article’s Creative Commons licence and your intended use is not permitted by statutory regulation or exceeds the permitted use, you will need to obtain permission directly from the copyright holder.

To view a copy of this licence, visit http://creativecommons.org/licenses/by/4.0/. 

